# Supplementary material for: A unipolar head gradient for high‐field MRI without encoding ambiguity
Source: Magn Reson Med. 2025 Sep 29;95(2):1266–78. doi: 10.1002/mrm.70098 (PMC12681299; doi:10.1002/mrm.70098)
Supplement: Supplementary file 1 — Figure S1. Operational region (OR) and peripheral nerve stimulation (PNS) threshold (TH) of different head z‐gradients. The data for the “Connectome 2.0” system were extracted from Figure 3 of Ramos et al.1 The data for the “Impulse” gradient were extracted from Figure 2 of Feinberg et al.3 using the curve labeled with “UBC+ERL.” As for both operation modes of the unipolar gradient of the present work, no PNS occurred, and no associated TH curve is shown. [file MRM-95-1266-s001.pdf]

Supplementary Information for

A unipolar head gradient for high-field MRI without encoding ambiguity

Markus Weiger[1], Johan Overweg, Franciszek Hennel[1],
Emily Louise Baadsvik[1], Samuel Bianchi[1], Oskar Björkqvist[1], Roger Luechinger[1], Jens Metzger[2], Eric Seth Michael[1], Thomas Schmid[1], Lauro Singenberger[1],
Urs Sturzenegger[3], Erik Oskam[3], Gerrit Vissers[4], Jos Koonen[4],
Wout Schuth[5], Jeroen Koeleman[5], Martino Borgo[5]
Klaas Paul Pruessmann[1]

[1]Institute for Biomedical Engineering, ETH Zurich and University of Zurich, Zurich, Switzerland

[2]Institute for Energy and Process Engineering, ETH Zurich, Zurich, Switzerland

[3]Philips AG, Zurich, Switzerland

[4]Philips Healthcare, Best, The Netherlands

[5]Futura Composites BV, Heerhugowaard, The Netherlands

PNS COMPARISON

To contextualise the absence of PNS observed for the presented unipolar gradient, its characteristics are compared here with two recently published head gradients with bipolar design. Figure S2 shows the operational region (OR) and PNS threshold (TH) of their z-gradients. Both the “Connectome 2.0” and the “Impulse” systems offer a larger nominal OR than the presented gradient. However, in both cases, the PNS threshold cuts away a substantial part of the respective OR. The ORs of the unipolar gradient run just below the thresholds of the other two systems, which is in agreement with the observed absence of PNS, and also indicates a practically useful set of specifications. Yet, due to the different ORs, the impact of unipolar versus bipolar design on PNS cannot be clarified here.

Notably, the z-gradients of the three systems offer different linearity, which in terms of field deviation in a sphere of 200 mm diameter is 11.7% for “Connectome 2.0” (1), 5.6% for “Impulse” (2), and 4.2% for the unipolar gradient. As for higher linearity lower PNS thresholds are expected, the higher threshold of the “Connectome 2.0” gradient may be explained by its reduced linearity.

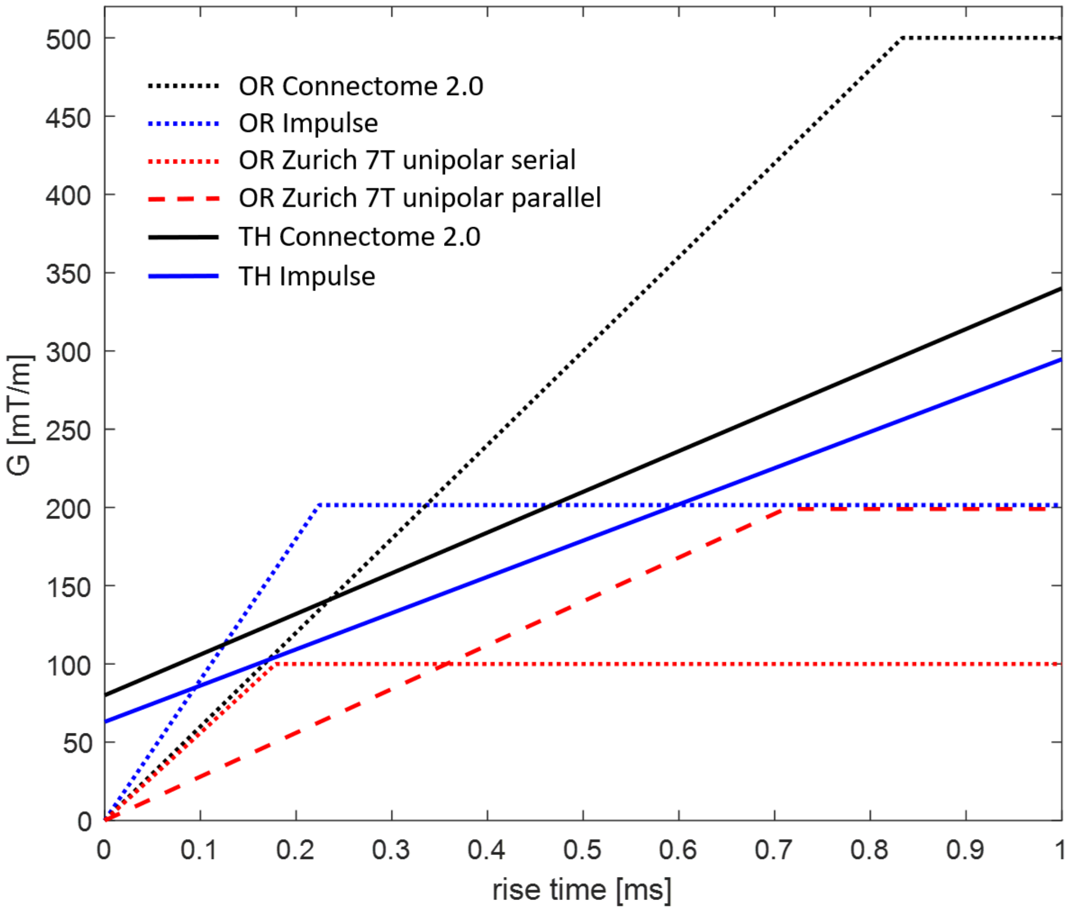

Figure S2: Operational region (OR) and PNS threshold (TH) of different head z-gradients. The data for the “Connectome 2.0” system was extracted from Figure 3 of Ref. (1). The data for the “Impulse” gradient was extracted from Figure 2 of Ref. (3) using the curve labelled with “UBC+ERL”. As for both operation modes of the unipolar gradient of the present work no PNS occurred, no associated TH curve is shown.

REFERENCES

1. Ramos-Llordén G, Lee H-H, Davids M, Dietz P, Krug A, Kirsch JE, Mahmutovic M, Müller A, Ma Y, Lee H, Maffei C, Yendiki A, Bilgic B, Park DJ, Tian Q, Clifford B, Lo W-C, Stocker S, Fischer J, Ruyters G, Roesler M, Potthast A, Benner T, Rummert E, Schuster R, Basser PJ, Witzel T, Wald LL, Rosen BR, Keil B, Huang SY. Ultra-high gradient connectomics and microstructure MRI scanner for imaging of human brain circuits across scales. Nature Biomedical Engineering 2025.
2. Davids M, Dietz P, Ruyters G, Roesler M, Klein V, Guerin B, Feinberg DA, Wald LL. Peripheral nerve stimulation informed design of a high-performance asymmetric head gradient coil. Magn Reson Med 2023;90:784-801.
3. Feinberg DA, Ma SJ, Walker E, Beckett AJS, Rattenbacher D, Rummert E, Dietz P, Davids M, Boulant N. Reassessment of peripheral nerve stimulation thresholds for the Impulse model-optimized asymmetric head gradient coil. Magn Reson Med 2025;94:1326-1338.
